# Prognostic Value of S100P Expression in Patients With Digestive System Cancers: A Meta-Analysis

**DOI:** 10.3389/fonc.2021.593728

**Published:** 2021-03-05

**Authors:** Bi-Xia Liu, Chao-Tao Tang, Xi-Jian Dai, Ling Zeng, Fei Cheng, Youxiang Chen, Chunyan Zeng

**Affiliations:** ^1^ Department of Gastroenterology, The First Affiliated Hospital of Nanchang University, Nanchang, China; ^2^ Department of Psychiatry, Faculty of Medicine, Chinese University of Hong Kong, Hong Kong, China; ^3^ Shenzhen Mental Health Centre, Shenzhen Kangning Hospital, Shenzhen, China

**Keywords:** S100 calcium-binding protein P (S100P), prognostic, meta-analysis, digestive system, cancers

## Abstract

**Background:**

Digestive system cancers (DSCs) are associated with high morbidity and mortality. S100P has been reported as a prognostic biomarker in DSCs, but its prognostic value remains controversial. Accordingly, we conducted a meta-analysis to investigate whether S100P is correlated with overall survival (OS) of patients with DSCs. The relationship between S100P and clinicopathological features was also evaluated.

**Methods:**

We systematically searched PubMed, Embase, Web of Science and Cochrane Library for eligible studies up to January 2020. In total, 16 publications with 1,925 patients were included.

**Results:**

S100P overexpression was associated with poor OS of patient with DSCs (HR=1.54, 95% CI: 1.14–2.08, P=0.005). When stratified by anatomic structure, S100P overexpression was associated with poor prognosis in non-gastrointestinal tract cancers (HR=1.98, 95% CI: 1.44–2.72, P<0.001) but not in gastrointestinal tract cancers (HR=1.09, 95% CI: 0.66–1.81, P=0.727). When stratified by tumor type, S100P overexpression predicted poor OS in cholangiocarcinoma (HR=2.14, 95% CI: 1.30–3.50, P=0.003) and hepatocellular carcinoma (HR=1.91, 95% CI: 1.22–2.99, P =0.005) but not in gastric cancer (HR=0.97, 95% CI: 0.65–1.45, P=0.872), colorectal cancer (HR=1.18, 95% CI: 0.32–4.41, P=0.807), gallbladder cancer (HR=1.40, 95% CI: 0.84-2.34, P=0.198), and pancreatic cancer (HR=1.92, 95% CI: 0.99–3.72, P=0.053). Furthermore, high S100P expression was significantly associated with distant metastasis (OR=3.58, P=0.044), advanced clinical stage (OR=2.03, P=0.041) and recurrence (OR=1.66, P=0.007).

**Conclusion:**

S100P might act as a prognostic indicator of non-gastrointestinal tract cancers.

## Introduction

Digestive system cancers (DSCs) are associated with high morbidity and mortality, which mainly consist of gastric cancer, colorectal cancer, hepatocellular carcinoma, esophageal cancer, pancreatic cancer, cholangiocarcinoma, and gallbladder cancer. Although some advances have been made in diagnosis and treatment in recent years, DSCs remain major threats for human health due to the high morbidity and mortality rates worldwide (1). Although numerous biomarkers involved in DSCs have been identified, sensitive imaging methods and biomarkers are scarce, and many DSC patients are identified at an advanced clinical stage, resulting in poor prognosis due to the high incidence of lymph node invasion, distant metastasis and local recurrence (2, 3). Moreover, patients with the same tumor characteristics, such as tumor size, tumor differentiation and clinical stage, may suffer from diverse clinical outcomes (4). Therefore, reliable new biomarkers are needed.

S100P is a member of the large family of S100 calcium-binding proteins, and it was initially isolated from human placenta (5, 6). S100P regulates a number of cellular processes through multiple signal pathways (7–11), and it is widely expressed in both normal and malignant tissues. In normal adult tissues, S100P exhibits the highest expression in the placenta and stomach (12). Overexpression of S100P has been detected in several tumors, including cholangiocarcinoma (11), colorectal cancer (13–15), lung cancer (16), pancreatic cancer (17), breast cancer (18), ovarian cancer (19), gastric cancer (20), and prostate carcinoma (12). S100P interacts with a number of signaling molecules both extracellularly and intracellularly (21, 22), and it has been demonstrated to mediate tumor growth, drug resistance, invasion, and metastasis (8, 13, 16, 17, 23–25). S100P is a potential biomarker for diagnosis and a potential target molecule for therapeutic intervention (8, 26, 27). Therefore, our primary hypothesis is that overexpression of S100P is associated with a poor prognosis in DSCs. The objective of the present meta-analysis study was to utilize existing literature to evaluate the prognostic value of S100P in DSCs.

## Methods

### Data Sources and Search Strategy

The PRISMA statement was followed in the systematic review and meta-analysis (28). We searched PubMed, Embase, Web of Science, and Cochrane Library up to January 2020. Studies that assessed the association between S100P expression and the survival outcome of DSCs patients were included in the meta-analysis. References reported in the included studies were also manually reviewed to identify potential missing studies in the initial search. The search terms were as follows: (S100P, S100 calcium-binding protein P, MIG9, or migration-inducing gene 9 protein) and (cancer, tumor, carcinoma, neoplasm, or malignancy) and (prognosis, prognostic, survival, outcome, or metastasis). No language restrictions were applied. Two investigators (Bi-Xia Liu and Chao-Tao Tang) searched the databases independently.

### Selection Criteria

Studies were eligible for inclusion based on the following criteria: (1) investigation of the relationship between S100P expression and overall survival (OS) or disease-free survival (DFS) in DSC patients; (2) data reported for hazard ratio (HR) and 95% confidence interval (95% CI) directly or indirectly (data allowing calculation of these values); (3) S100P expression was detected in the tumor tissues of DSC patients; (4) patients were divided into high and low expression subgroups according to the cut-off value of S100P; (5) published in full text; and (6) human studies. The exclusion criteria were as follows: (i) insufficient data; (ii) previously published or smaller sample size studies were excluded after identification of duplicate publications or duplicate data; and (iii) reviews, letters, case reports, conference abstracts, or laboratory studies. Ethical approval and patient consent were not required for this meta-analysis.

### Data Extraction and Quality Assessment

Two investigators (Bi-Xia Liu and Chao-Tao Tang) independently evaluated and extracted all the essential data from enrolled articles. Any disagreements were resolved through discussion. The following information was extracted from the included studies: first author’s name; publication year; number of cases; gender; age; country; cancer type; test method; cut-off value for high expression of S100P; outcome; HR and corresponding 95% CI for OS and DFS; and clinicopathological characteristics, including differentiation, lymph node metastasis, distant metastasis, T stage, clinical stage, vascular invasion, and recurrence. The quality of the included studies was assessed according to the Newcastle-Ottawa Scale (NOS) (29). The NOS contains eight items and categorized into three dimensions as follows: selection (0–4 points), comparability (0–2 points) and outcome assessment (0–3 points). The NOS score ranges from 0 to 9, and studies with NOS scores ≥6 are considered high quality.

### Statistical Analysis

Statistical analyses were conducted with Stata MP16 Software (STATA Corp., College Station, Texas, USA). The HRs and 95% CIs were utilized to assess the prognostic value of S100P expression on the survival of patients with DSCs. HR >1 with p-value <0.05 indicated a poor prognosis of S100P overexpression in DSC patients. The odds ratios (ORs) with corresponding 95% CIs were used to analyze the association between the S100P expression level and the clinicopathological parameters. The HRs and 95% CIs were obtained directly from the published studies or extracted from the Kaplan–Meier survival curves by Engauge Digitizer version 12.1. Heterogeneity among the enrolled studies was quantitated with I^2^ statistics. The random-effect model was applied if heterogeneity was present (I^2^ ≥ 50% or P ≤ 0.1), and if heterogeneity was not present, the fixed-effect model was used. Subgroup analysis, as stratified by anatomic structure, cancer type, sample size, and study region, was performed. Sensitivity analysis was conducted to assess the reliability of the results. Meta-aggression analysis was conducted to identify the source of heterogeneity. Publication bias was measured by Begg’s test and Egger’s test. P-value <0.05 was considered statistically significant.

## Results

### Study Selection

**Figure 1:**
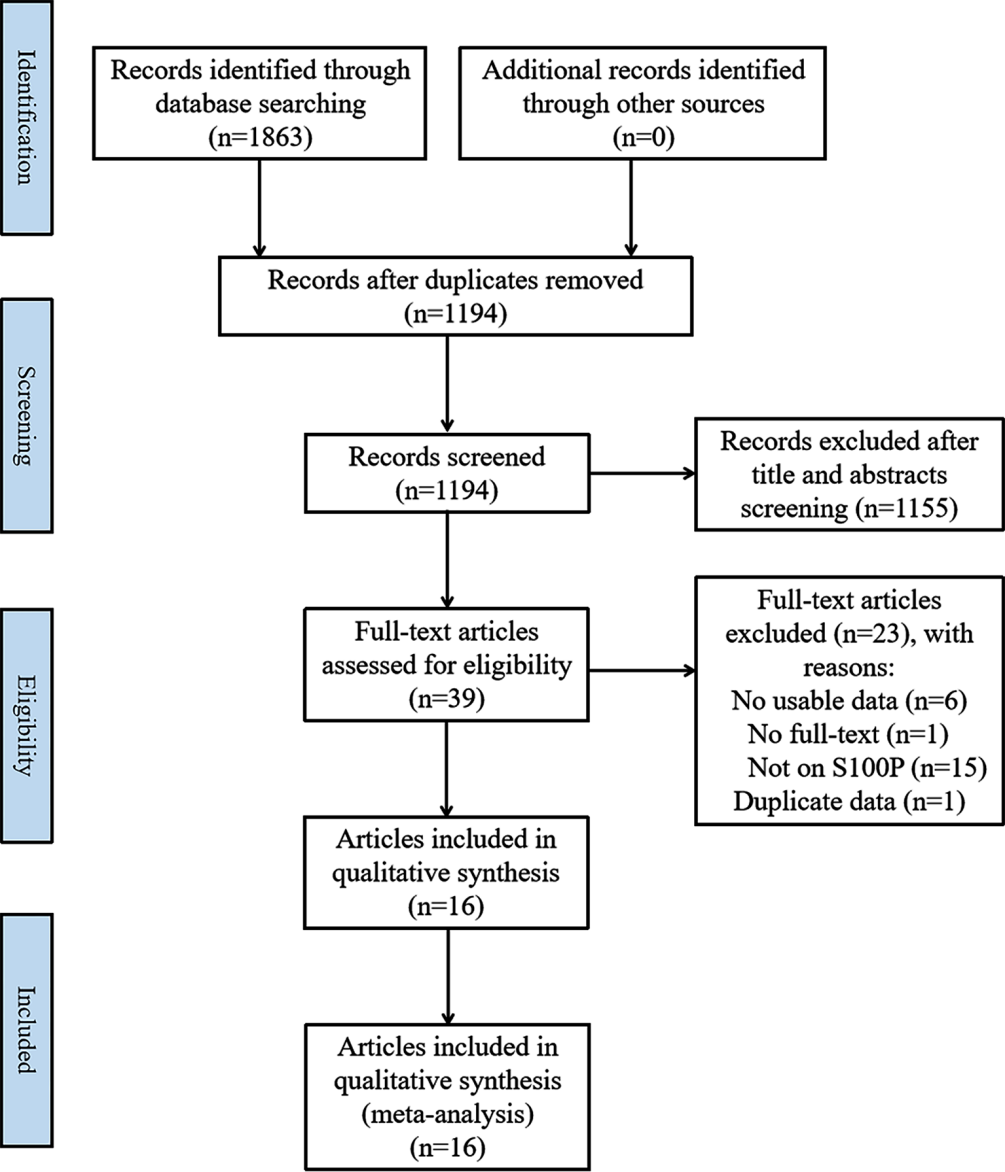
Flow diagram of the search and selection process.

The literature search and selection process are shown in **Figure 1**. In total, 1,863 articles were retrieved in the initial search. After removing the duplicate articles, 1,194 articles were entered into further screening. In total, 1,155 articles were discarded after careful review of the title and abstract. Subsequently, the full texts of the remaining 39 articles were evaluated, and 23 articles were excluded due to the following reasons: insufficient data in six articles; S100P not the main focus in 15 articles; unavailable full-text in one article; and overlapped full-text in one article. Finally, the remaining 16 articles (11, 13, 20, 23, 27, 30–40), comprising 1,925 patients, were enrolled in the current meta-analysis.

### Characteristics of Included Studies

The basic characteristics of the included studies are shown in **Table 1**. The included studies were published between 2009 and 2019. Specifically, one study by Aishima et al. investigated two cancer subtypes and was marked as Aishima et al. (1) and Aishima et al. (2) (38). Therefore, 16 articles with 17 studies were included. Among the included articles, one was published in Chinese, and the others were published in English. The patients were enrolled from the following five countries: China, Japan, Thailand, Portugal, and Italy. Four studies reported on patients with gastric cancer (GC) (20, 30, 39, 40), and three studies focused on patients with colon cancer (CC) or colorectal cancer (CRC) (13, 23, 27). Two studies reported on patients with cholangiocarcinoma (CCA) (11, 34), and four studies reported on patients with intrahepatic cholangiocarcinoma (ICC) (32, 37, 38). One study reported on patients with extrahepatic cholangiocarcinoma (ECC) or extrahepatic bile duct carcinoma (EHBDC) (36), and one study reported on patients with hepatocellular carcinoma (HCC) (35). One study reported on patients with gallbladder cancer (GBC) (33), and one study reported on patients with pancreatic cancer (PC) (31). Because CCA originates from the epithelium of the bile duct and is further classified into ICC and ECC (41), the CCA, ICC, and EHBDC were categorized into the same group in the following subgroup analysis. S100P expression in tumor tissues was detected by IHC in 15 studies, RT-PCR in one study and western blotting (WB) in one study.

**Table 1:** Baseline characteristics of studies included in the meta-analysis.

Author	Year	Country	Cancer type	Case number/male (n)	Age (years) Median/mean (range)	Test method	cut-off of high expression	High S100P expression: n (%)	Outcome	HR (95% CI) for OS	HR availability	NOS score
Carneiro et al. (30)	2019	Portugal	GC	318/181	Range:32–95	IHC	stained graded:3+	231(72.6%)	OS	1.09(0.78–1.52)	Indirectly	8
Ge et al. (20)	2013	China	GC	156/NR	NR	RT-PCR	Median value	82(52.6%)	OS	1.80(1.12–3.51)	Indirectly	6
Zhao et al. (39)	2010	China	GC	121/86	Mean:58 Range:26–82	IHC	stained graded ≥2+	64(52.9%)	OS	0.77(0.47–1.26)	Indirectly	6
Jia et al. (40)	2009	China	GC	93/NR	NR	IHC	stained cells >5%	44 (47.3%)	OS	0.62(0.38–0.93)	Indirectly	6
Shen et al. (23)	2016	China	CC	125/49	≤60:71; >60:54	IHC	stained graded≥2+	55(44%)	OS	3.33(2.11–5.26)	Directly	7
Dong et al. (13)	2014	China	CRC	91/39	Mean:60.09 Range:34–81	IHC	H score≥102	34(37.4%)	OS	0.66(0.25–1.74)	Indirectly	7
Wang et al. (27)	2012	China	CRC	96/61	Mean:62.4 range: 28–92	WB	NR	63(65.6%)	OS	0.55(0.13–2.28)	Indirectly	7
Wu et al. (11)	2016	Thailand	CCA	78/50	< 60:50; ≥ 60:28	IHC	stained graded≥3+	46(59%)	OS	3.37(1.60–7.12)	Indirectly	6
Sato et al. (34)	2013	Japan	CCA	33/21	Mean:73	IHC	stained graded≥1+	29(88%)	OS	0.45(0.13–1.56)	Indirectly	6
Sarcognato et al. (32)	2019	Italy	ICC	61/26	Mean:67 Range:35–82	IHC	stained graded as 2+	12(19.7%)	OS	1.66(1.08–2.55)	Directly	6
Tsai et al. (37)	2012	China	ICC	112/60	Mean:60.8 Range:30–87	IHC	stained cells >1%	59(52.7%)	OS	1.62(1.14–2.30)	Indirectly	6
Aishima et al. (1) (38)	2011	Japan	Perihilar ICC	41/NR	NR	IHC	stained graded ≥ 2+	28(68.3%)	OS	1.71(0.65–4.51)	Indirectly	6
Aishima et al. (2) (38)	2011	Japan	Peripheral ICC	69/NR	NR	IHC	stained graded ≥2+	8(11.6%)	OS	4.78(2.77–8.26)	Indirectly	6
Kawashima et al. (36)	2013	Japan	EHBDC	55/42	< 65:21; ≥ 65:34	IHC	Stained cells >1%	40(72.7%)	OS	8.51(1.18–64.8)	Directly	7
Yuan et al. (35)	2013	China	HCC	305/239	Mean:55.09 Range:15–88	IHC	stained cells>1%	173(56.7%)	OS	1.91(1.22–2.99)	Directly	6
Li et al. (33)	2016	China	GBC	81/27	Mean:66.8	IHC	stained cells >5%	50(61.7%)	OS	1.40(0.84–2.34)	Indirectly	6
Nakayama et al. (31)	2019	Japan	PC	90/51	Mean:66 Range:36–85	IHC	NR	45 (50%)	OS, DFS	1.92 (0.99–3.72)	Indirectly	6

GC, gastric cancer; CC, Colon Cancer; CRC, colorectal cancer; CCA, Cholangiocarcinoma; ICC, Intrahepatic cholangiocarcinoma; EHBDC, extrahepatic bile duct carcinoma; HCC, hepatocellular carcinoma; GBC, Gallbladder cancer; PC, pancreatic cancer; IHC, immunohistochemistry; RT-PCR, real-time polymerase chain reaction; WB, Western blotting; OS, overall survival; DFS, disease-free survival; HR, hazard ratio; CI, confidence interval; NR, not reported.

The NOS scores of the included studies ranged from 6 to 8, indicating high-quality of the included studies (**Table 2**).

**Table 2:** Summary of Newcastle-Ottawa quality assessment scale.

Newcastle-Ottawa scale category				
	Selection	Comparability	Outcome	Total
Carneiro et al. (30)	****	*	***	8
Nakayama et al. (31)	****	/	**	6
Sarcognato et al. (32)	****	/	**	6
Wu et al. (11)	****	/	**	6
Shen et al. (23)	****	*	**	7
Li et al. (33)	****	/	**	6
Dong et al. (13)	****	*	**	7
Sato et al. (34)	****	/	**	6
Yuan et al. (35)	****	/	**	6
Ge et al. (20)	****	/	**	6
Kawashima et al. (36)	****	*	**	7
WANG, 2012	****	*	**	7
Tsai et al. (37)	****	/	**	6
Aishima et al. (1) (38)	****	/	**	6
Aishima et al. (2) (38)	****	/	**	6
Zhao et al. (39)	****	/	**	6
Jia et al. (40)	****	/	**	6

Score with an asterisk, *: one point; **: two points; ***: three points; ****: four points.

### Correlation of High S100P Expression With OS in Digestive System Cancers

**Figure 2:**
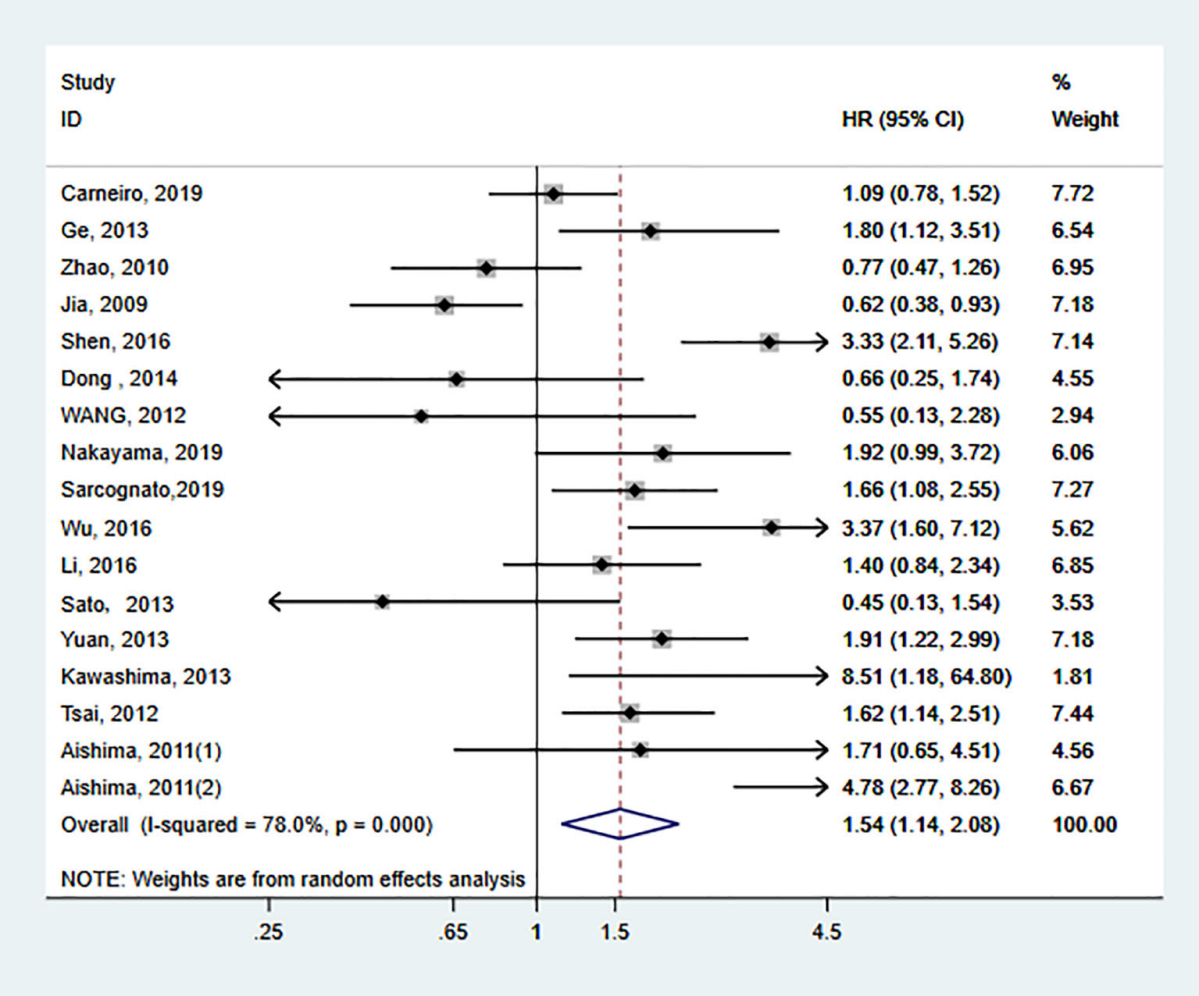
Forest plot for the relationship between S100P expression and overall survival (OS).

**Figure 3:**
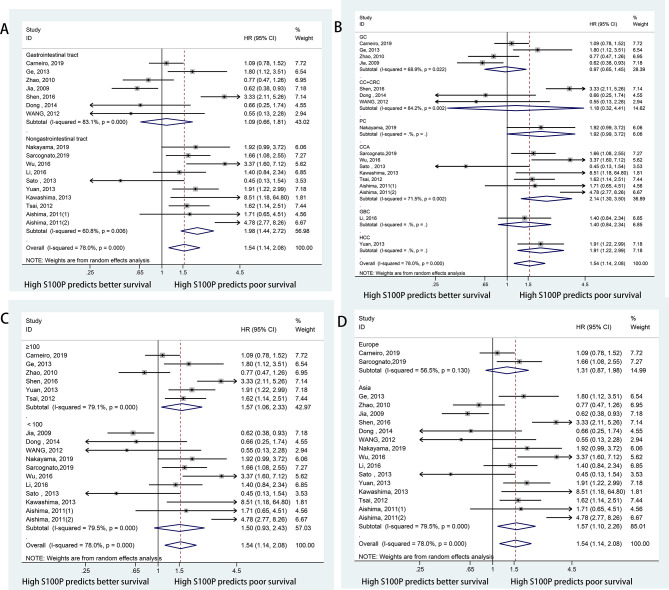
Forest plots for the association between S100P expression and OS categorized by different subgroups: **(A)** Subgroup analysis of OS by anatomic structure; **(B)** Subgroup analysis of OS by cancer type; **(C)** Subgroup analysis of OS by sample size; **(D)** Subgroup analysis of OS by study region.

Data for the association between S100P expression and OS were extracted from all the included 17 studies (11, 13, 20, 23, 27, 30–40). The heterogeneity was significant among these studies (I^2^ = 78.0%, P<0.001); therefore, the random-effects model was used. The pooled result revealed that high S100P expression was significantly associated with poor OS of DSC patients (n=17, pooled HR=1.54, 95% CI=1.14–2.08, P=0.005) (**Table 3** and **Figure 2**). In addition, subgroup analysis was performed for further investigation. When the cancers were stratified by anatomic structure, the data showed that high S100P expression remained a significant factor of poor OS in patients with non-gastrointestinal tract cancer (n=10, HR=1.98, 95% CI= 1.44–2.72, P<0.001) but not in patients with gastrointestinal tract cancer (n=7, HR=1.09, 95% CI=0.66-1.81, P=0.727; **Table 3** and **Figure 3A**). When the subgroup analysis was conducted by cancer type, the data revealed that the high S100P level significantly led to poor OS in patients with CCA (n=7, HR=2.14, 95% CI=1.30–3.50, P=0.003) and in patients with HCC (n=1, HR=1.91, 95% CI=1.22–2.99, P=0.005) but not in patients with GC (n=4, HR=0.97, 95% CI=0.65–1.45, P=0.872), CRC (n=3, HR=1.18, 95% CI=0.32–4.41, P=0.807), GBC (n=1, HR=1.40, 95% CI=0.84, 2.34, P=0.198) and PC (n=1, HR=1.92, 95% CI=0.99–3.72, P=0.053; **Table 3** and **Figure 3B**). Furthermore, S100P expression was also a prognostic factor of poor OS in studies with large sample size (n=6, HR=1.57, 95% CI=1.06–2.33, P=0.024) and in an Asian population (n=15, HR=1.57, 95% CI=1.10–2.26, P=0.014) but not in studies with small sample size (n=11, HR=1.50, 95% CI=0.93–2.43, P=0.098) and in an European population (n=2, HR=1.31, 95% CI=0.87–1.98, P=0.190; **Table 3** and **Figures 3C, D**). As the detect methods and the cut-off value of S100P in studies included in meta-analysis were not consistent, we further performed subgroup analysis according to detect methods and cut-off value. The results showed that S100P expression was a prognostic factor of poor OS in studies when S100P detected by IHC (n=15, HR=1.57, 95% CI=1.13–2.18, P=0.007) and detected by RT-PCR (n=1, HR=1.80, 95% CI=1.02–3.19, P=0.044) but not in studies when S100P detected by WB (n=1, HR=0.55, 95% CI=0.13–2.30, P=0.413; **Table 3** and **Supplementary Figure 1**). Furthermore, S100P expression was also a prognostic factor of poor OS in studies when the cut-off values were stained grade 2+ (n=5, HR=2.04, 95% CI=1.06–3.93, P=0.032) and stained cells 1% (n=3, HR=1.84, 95% CI=1.27–2.67, P=0.001; **Table 3** and **Supplementary Figure 2**). The DFS was not analyzed because only one study was eligible.

**Table 3:** Subgroup analysis of the HRs of overall survival of patients with high S100P expression level.

Subgroup	No. of studies	No. of patients	HR (95% CI)	P value	Heterogeneity			Meta- regression, p value
					I^2^ (%)	P value	Model	
**Total**	17	1925	1.54(1.14, 2.08)	0.005	78.0	<0.001	random
**Anatomic structure**								0.758
Gastrointestinal tract	7	1,000	1.09 (0.66, 1.81)	0.727	83.1	<0.001	random	
Non-gastrointestinal tract	10	925	1.98 (1.44, 2.72)	<0.001	60.8	0.006	random	
**Tumor type**								0.925
Gastric cancer	4	688	0.97 (0.65, 1.45)	0.872	68.9	0.022	random	
Colon cancer + Colorectal cancer	3	312	1.18 (0.32, 4.41)	0.807	84.2	0.002	random	
Cholangiocarcinoma	7	449	2.14 (1.30, 3.50)	0.003	71.5	0.002	random	
Hepatocellular carcinoma	1	305	1.91 (1.22, 2.99)	0.005	–	–	–	
Gallbladder cancer	1	81	1.40 (0.84, 2.34)	0.198	–	–	–	
Pancreatic cancer	1	90	1.92 (0.99, 3.72)	0.053	–	–	–	
**Sample size**								0.456
≥100	6	1,137	1.57 (1.06, 2.33)	0.024	79.1	<0.001	random	
<100	11	788	1.50 (0.93, 2.43)	0.098	79.5	<0.001	random	
**Study region**								0.319
Europe	2	1,546	1.31 (0.87, 1.98)	0.190	56.5	0.130	random	
Asia	15	379	1.57 (1.10, 2.26)	0.014	79.5	<0.001	random	
**Detect method**								0.773
IHC	15	1,673	1.57 (1.13, 2.18)	0.007	80.1	<0.001	random	
RT-PCR	1	156	1.80 (1.02, 3.19)	0.044	–	–	–	
WB	1	96	0.55 (0.13, 2.30)	0.413	–	–	–	
**Cut-off value**								0.310
Stained grade 3+	2	396	1.82 (0.61, 5.48)	0.286	86.3	0.007	random	
Stained grade 2+	5	417	2.04 (1.06, 3.93)	0.032	86.6	<0.001	random	
Stained cells 5%	2	174	0.92 (0.42, 2.05)	0.843	81.8	0.019	random	
Stained cells 1%	3	472	1.84 (1.27, 2.67)	0.001	24.6	0.266	fixed	
NR	3	342	1.62 (0.99, 2.65)	0.054	21.5	0.280	fixed	
Others	2	124	0.57(0.27, 1.22)	0.149	0.0	0.633	fixed	

CI, confidence interval; HR, hazard ratio; NR, no report; IHC, immunohistochemistry; RT-PCR, real-time polymerase chain reaction; WB, Western blotting; No., Number.

### Correlation of S100P and Clinicopathological Characteristics

The relationships between S100P expression and clinicopathological features are illustrated in **Table 4**. No significant heterogeneity was observed among studies regarding differentiation grade (I^2^ = 0, P=0.683), T stage (I^2^ = 0, P=0.399), vascular invasion (I^2^ = 44.8, P=0.163) and recurrence (I^2^ = 48.3, P=0.144); therefore, the fixed-effects model was applied. Nevertheless, significant heterogeneity was detected among studies regarding gender (I^2^ = 51.4, P=0.036), lymph node metastasis (I^2^ = 69.6, P=0.001), distant metastasis (I^2^ = 81.3, P=0.001) and clinical stage (I^2^ = 77.0, P=0.001); thus, the random effects model was used. We observed that high S100P expression was significantly correlated with certain phenotypes of tumor aggressiveness, such as distant metastasis (OR=3.58, 95% CI: 1.04–12.36, P=0.044), advanced clinical stage (OR=2.03; 95% CI=1.03–4.01; P=0.041) and recurrence (OR=1.66; 95% CI=1.15–2.38; P=0.007). This finding indicated that S100P may promote tumor invasion and recurrence. However, no significance correlation was found between S100P expression and gender (OR=0.91, 95% CI: 0.63–1.32, P=0.617), differentiation grade (OR=1.09, 95% CI: 0.75–1.57, P=0.652), lymph node metastasis (OR=1.66, 95% CI: 0.93–2.97, P=0.084), T stage (OR=0.89, 95% CI: 0.59–1.36, P=0.598), or vascular invasion (OR=0.99, 95% CI: 0.63–1.57, P=0.978).

**Table 4:** Pooled ORs for the relationship between S100P expression levels and clinicopathological parameters.

Clinicopathological feature	Number of studies	Number of patients	OR (95% CI)	P	Heterogeneity		
					I^2^ (%)	P	Model
Gender (male vs female)	9	1,301	0.91 (0.63–1.32)	0.617	51.4	0.036	Random
Differentiation (poor vs moderate/well)	9	713	1.09 (0.75–1.57)	0.652	0.0	0.683	Fixed
Lymph node metastasis (yes vs no)	10	1,043	1.66 (0.93–2.97)	0.084	69.6	0.001	Random
Distant metastasis (yes vs no)	4	405	3.58 (1.04–12.36)	0.044	81.3	0.001	Random
T stage (T3/T4 vs T1/T2)	3	475	0.89 (0.59–1.36)	0.598	0.0	0.399	Fixed
Clinical stage (III/IV vs I/II)	7	820	2.03 (1.03–4.01)	0.041	77.0	0.001	Random
Vascular invasion (Present vs Absent)	3	423	0.99 (0.63–1.57)	0.978	44.8	0.163	Fixed
Recurrence (yes vs no)	3	506	1.66 (1.15–2.38)	0.007	48.3	0.144	Fixed

We also analyzed the correlation between S100P expression and the clinicopathological factors in GC, CRC, and CCA. HCC, GBC, and PC were not analyzed because only one study was included for the three cancers. The results of pooled OR and 95% CI showed that there was significant correlation between S100P expression and gender in GC (P=0.019) but that there was no significant correlation between S100P expression and lymph node metastasis (P=0.556). In addition, there was no significant association between S100P expression and gender (P=0.349), differentiation grade (P=0.926), lymph node metastasis (P=0.113) or clinical stage (P=0.274) in patients with CRC. For patients with CCA, there was significant correlation between S100P expression and lymph node metastasis (P<0.001), but there was no significant correlation between S100P expression and gender (P=0.671) or differentiation grade (P=0.105) (**Supplementary Materials: Table S1**).

### Sensitivity Analysis and Meta-Regression Analysis

**Figure 4:**
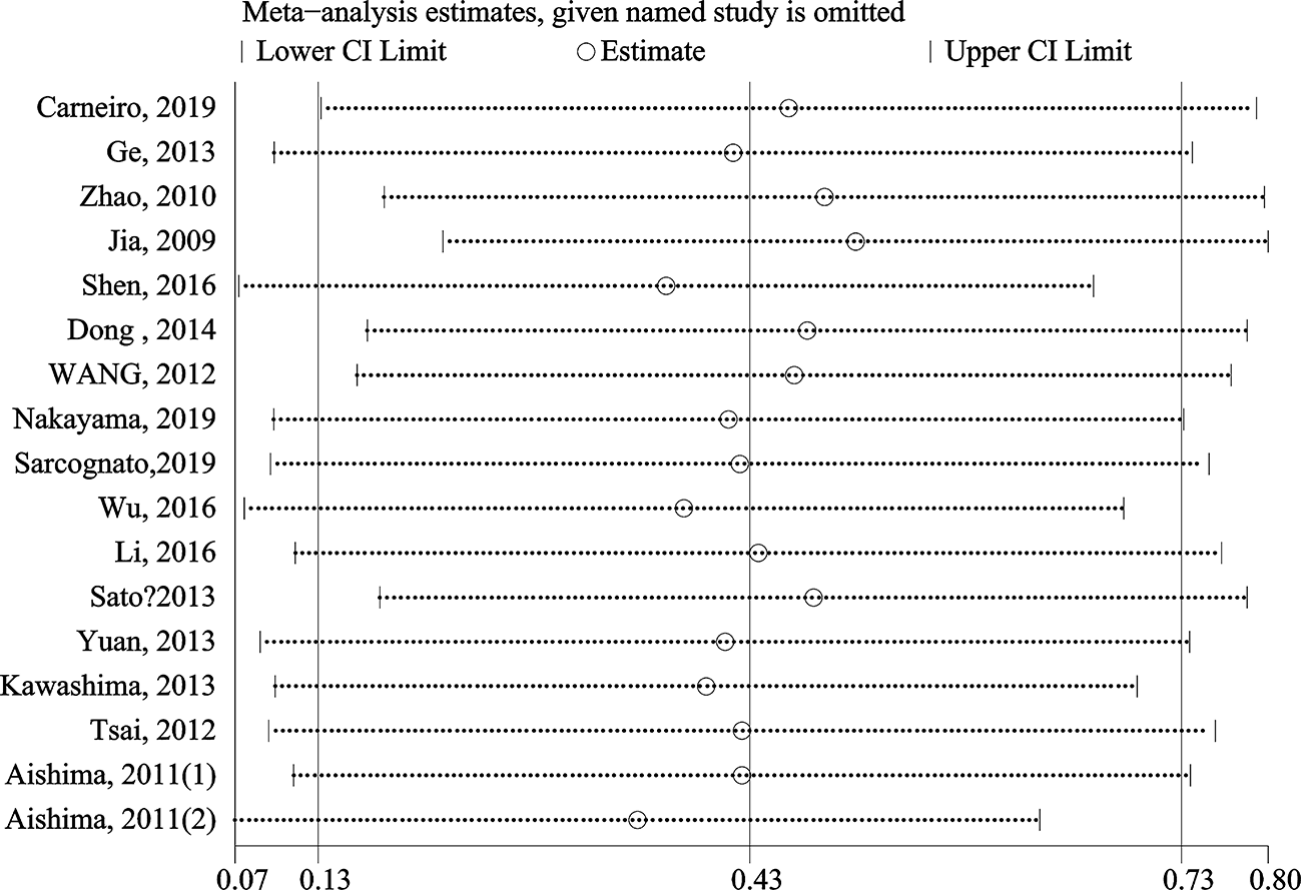
Sensitivity analysis of S100P expression for OS.

The stability and reliability of pooled HRs for OS were evaluated by sensitivity analysis. The results demonstrated that the conclusions were stable and reliable because the pooled HRs were not significantly affected by any individual study (**Figure 4**). Meta-regression analysis showed that the anatomic structure (P=0.758), cancer type (P=0.925), sample size (P=0.456), study region (P=0.319), detect method (P=0.773) and cut-off value (P=0.310) did not significantly contribute to the heterogeneity of OS (**Table 3**).

### Publication Bias

**Figure 5:**
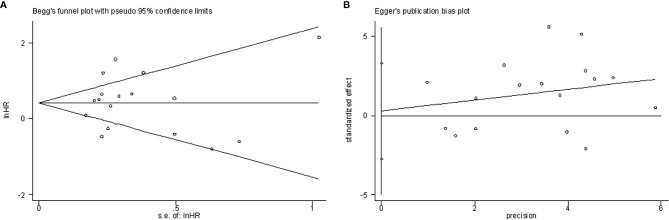
Publication bias examination. **(A)** Begg’s funnel plots assessing the publication bias for OS (p=0.650); **(B)** Egger’s test assessing the publication bias for OS (p=0.846).

Begg’s funnel plots and Egger’s test were used to estimate the potential publication bias. The results showed that there was no significant publication bias for OS according to Begg’s test (P=0.650, **Figure 5A**) and Egger’s test (P=0.846, **Figure 5B**).

## Discussion

DSCs are a diverse group of tumors and have poor prognosis due to the advanced stage at the time of initial diagnosis. Despite that many achievements have been made in clinical and experimental studies, sensitive prognostic biomarkers are scarce, and reliable biomarkers are needed. Evidence has indicated that S100P overexpression is associated with poor prognosis in patients with some cancers (16, 17, 20, 23, 24), but conflicting results have also been reported, indicating that S100P may enhance the chemosensitivity of GC (42). Therefore, it remains unknown whether S100P can serve as a biomarker to predict the prognosis of DSC patients. To evaluate the correlation between S100P expression and OS in DSC patients, 17 studies in 16 publications with 1,925 patients were enrolled in the present meta-analysis (11, 13, 20, 23, 27, 30–40). In addition, we also explored the relationship between S100P expression and clinicopathological characteristics of DSCs.

Subgroup analyses by anatomic structure and tumor type were separately performed in DSC patients due to different TNM stage and prognosis among various tumor types. First, the prognostic value of S100P expression in all types of DSCs was evaluated. The results demonstrated that S100P overexpression was associated with poor OS in patients with DSCs, and higher predictive value of S100P was observed in non-gastrointestinal tract tumors compared to gastrointestinal tract tumors according to subgroup analysis. When subgroup analysis was performed by tumor type, the results revealed that S100P overexpression was associated with poor OS in CCA and HCC. However, high S100P expression was not related to OS in gastric cancer, colorectal cancer, gallbladder cancer, and pancreatic cancer. Furthermore, when subgroup analysis was performed by sample size and study region, the pooled results were also significant in studies with large sample size and based on an Asian population but not in studies with small sample size and based on a European population. Sensitivity analysis, meta-regression analysis and publication bias tests suggested that these results were stable and credible.

Despite the robust results in the present meta-analysis, these findings should be interpreted with caution. First, the heterogeneity among the included studies was significant in our meta-analysis, even in the subgroup analyses. We analyzed that the significant heterogeneity may be caused by patient features, cancer types, ethnicity, literature quality, the detect methods and the cut-off values of S100P. So we performed a meta-regression analysis to identify the source of heterogeneity. However, none of these confounding factors completely explained the heterogeneity. Our meta-analysis showed that the prognostic value of high S100P expression varied with tumor types in DSC patients, which may be due to several mechanisms. First, S100P expression levels are varied in different tissues. Among the normal tissues, the highest S100P mRNA levels were detected in the placenta and esophagus. Moderate signals were detected in the stomach, duodenum, large intestine, prostate and leukocytes. At the protein level, the highest levels of S100P were detected in the placenta and stomach. Among the tumor tissues, however, S100P was most prevalent in gastric tumors (12). For those tissues with high S100P expression, S100P may play an important role in maintaining the normal status of cells. For those tissues with weak or no S100P expression with normal status, however, the elevated S100P expression level may have a relatively adverse effect on the cells. Second, it has been reported that S100P is involved in various cellular functions (5, 7, 8, 43), indicating that it is a target protein or mediator protein for multiple signaling pathways (33, 44–46). These signaling pathways may play different roles in carcinogenesis in different tumors. Carneiro et al. (30) reported that S100P has a dual role in gastric cancer, acting as an oncogenic factor in the context of E-cadherin loss and as a tumor suppressor in a functional E-cadherin setting. Third, because only one article on hepatocellular carcinoma, gallbladder cancer and pancreatic cancer was included, the pooled HRs were weakly effective. Therefore, more studies are needed to investigate the functions of S100P in different cancers to explain the prognostic value of S100P.

Regarding the clinicopathological characteristics, our results indicated that higher expression levels of S100P indicated a greater possibility of distant metastasis, advance clinical stage and recurrence. Considering that the biology, pathology, clinical courses, and treatments vary among different types of DSCs, we also assessed the relationship between S100P expression and the clinicopathological characteristics in gastric cancer, colorectal cancer and cholangiocarcinoma. High S100P expression was not evaluated in hepatocellular carcinoma, gallbladder cancer, and pancreatic cancer due to limited data on the clinicopathological features. Furthermore, no significant correlation between S100P expression and clinicopathological characteristics in colorectal cancer was found, which may be due to the limited enrolled studies. S100P expression was significantly higher in females than in males in gastric cancer. Furthermore, increased S100P expression in cholangiocarcinoma was significantly associated with lymph node metastasis, which is consistent with the pooled results of OS in cholangiocarcinoma, suggesting that S100P overexpression may promote invasion and metastasis.

Our data suggested that S100P may be a potential reliable prognostic biomarker in some DSCs. The findings of the present study broadened and expanded the current understanding of S100P. However, there are several limitations that should be addressed. First, the number of 16 included publications for the topic of the digestive system seems small, especially when divided into six subgroups. And some report with small sample sizes. Due to the small number of included publications, our meta-analysis focus on the prognostic value of S100P in the digestive system which seems to be too broad. These may reduce the reliability of the results. But the sensitivity analysis, meta-regression analysis and publication bias tests suggested that these results were stable and credible in our study. In the future, more studies with large sample sizes and low incidence tumors are needed. Second, some of the HRs were calculated from Kaplan-Meier survival curves rather than directly obtained from the primary data. Although the data of HRs are extracted by accepted methods, it would be better to obtain HRs directly from the literature. Third, patients enrolled in this meta-analysis were mostly from Asia, which may lead to selection bias. Fourth, although the meta-regression analysis showed that the detect method and cut-off value did not significantly contribute to the heterogeneity. But due to the small number of literatures in some subgroups, the actual situation may not be fully reflected. So we suspected that the detect methods and the cut-off values of S100P in studies included in meta-analysis were not consistent, which may relate to the heterogeneity of these studies. Unified detect methods and cut-off value should be used in the future study, and standardized conversion of cut-off values of different detection methods should be carried out, which is helpful to reduce heterogeneity in different studies and increase the reliability of the results.

## Conclusions

In summary, our meta-analysis demonstrated that S100P overexpression was associated with poor OS of DSC patients. The prognostic value of S100P expression was significant for the OS of patients with cholangiocarcinoma and hepatocellular carcinoma but not for patients with gastric cancer, colorectal cancer, gallbladder cancer, and pancreatic cancer. When stratified by anatomic structure, S100P overexpression was associated with poor prognosis in non-gastrointestinal tract cancers but not in gastrointestinal tract cancers. Furthermore, significant correlation was observed between S100P expression and some phenotypes of tumor aggressiveness, such as distant metastasis, advanced clinical stage, and recurrence. These results indicated that S100P may be an effective factor of poor prognosis in some digestive system cancers, especially in non-gastrointestinal tract cancers. Nevertheless, as the study had several limitations, further large-scale, well-designed studies are needed to confirm our results.

## Data Availability Statement

The raw data supporting the conclusions of this article will be made available by the authors, without undue reservation.

## Author Contributions

B-XL, C-TT, and X-JD were responsible for study design. B-XL and C-TT were responsible for literature search. B-XL and C-TT were responsible for data extraction. X-JD, LZ, and FC were responsible for data analysis. B-XL and C-TT were responsible for drafting the manuscript. CZ and YC approved the final version of the manuscript. All authors contributed to the article and approved the submitted version.

## Funding

This study was supported by grants from the Key Foundation of Jiangxi Provincial Department of Science and Technology (grant No. 20202ACBL206018), the Foundation of Jiangxi Provincial Department of Science and Technology (grant No. 20202BAB206051), the Foundation of Jiangxi Provincial Department of Science and Technology (grant No. 20201ZDG02007), the National Natural Science Foundation of China (grant No. 81701678), the Science and Technology Foundation of Jiangxi Provincial Health Commission (grant No. 20191098), and the China Postdoctoral Science Foundation (grant No, 2020M670052). Sanming Project of Medicine in Shenzhen (grant No, SZSM201812052).

## Conflict of Interest

The authors declare that the research was conducted in the absence of any commercial or financial relationships that could be construed as a potential conflict of interest.

## Supplementary Material

Supplementary Figure 1 Forest plots for the association between S100P expression and OS categorized by detect methods.

Supplementary Figure 2 Forest plots for the association between S100P expression and OS categorized by cut-off value.
