# Supplementary figures and images for: Prognostic Value of S100P Expression in Patients With Digestive System Cancers: A Meta-Analysis
Source: Front Oncol. 2021 Mar 5;11:593728. doi: 10.3389/fonc.2021.593728 (PMC7973272; doi:10.3389/fonc.2021.593728)

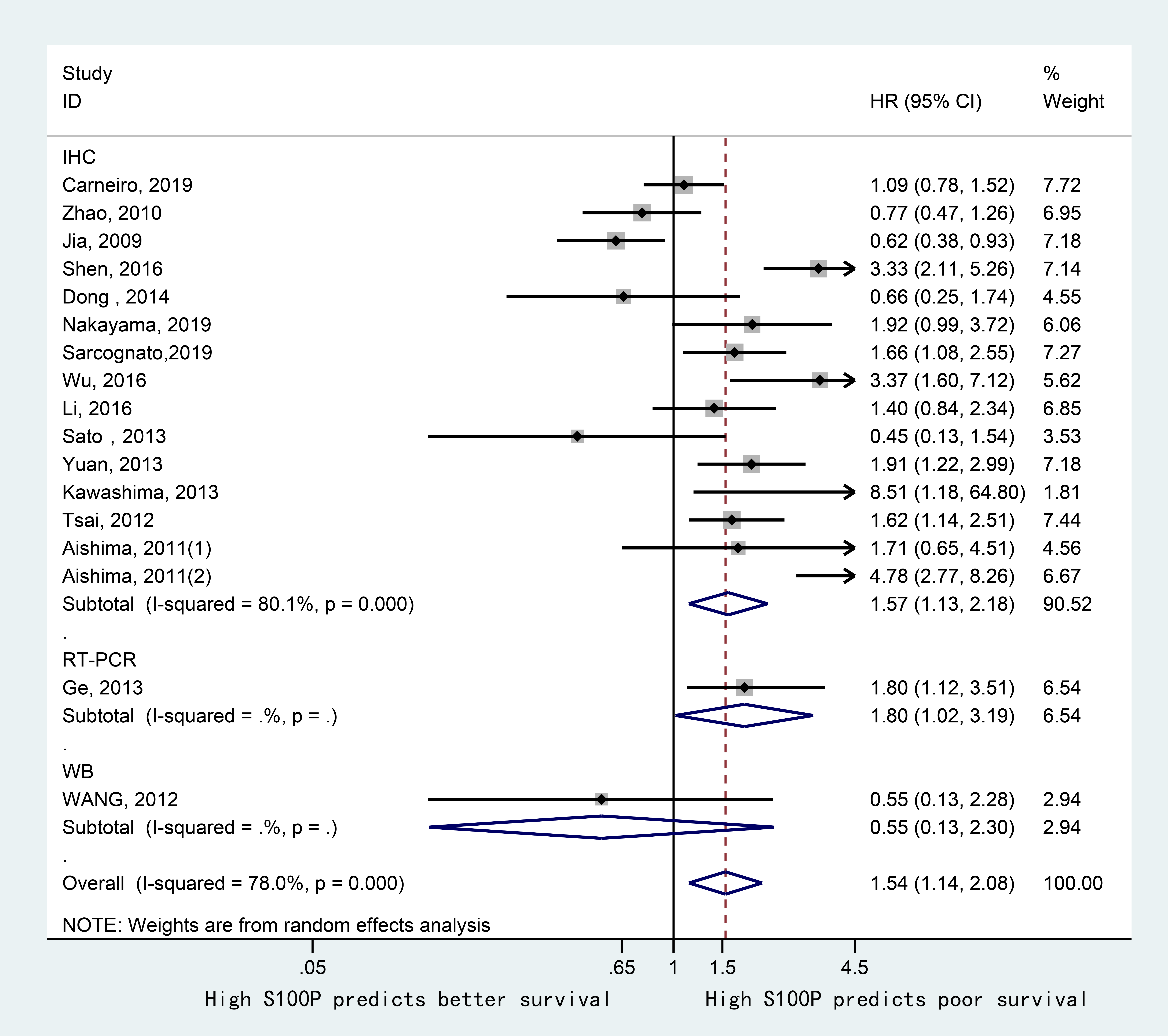

Supplement: Supplementary Figure 1 — Forest plots for the association between S100P expression and OS categorized by detect methods. [file Image_1.tif]

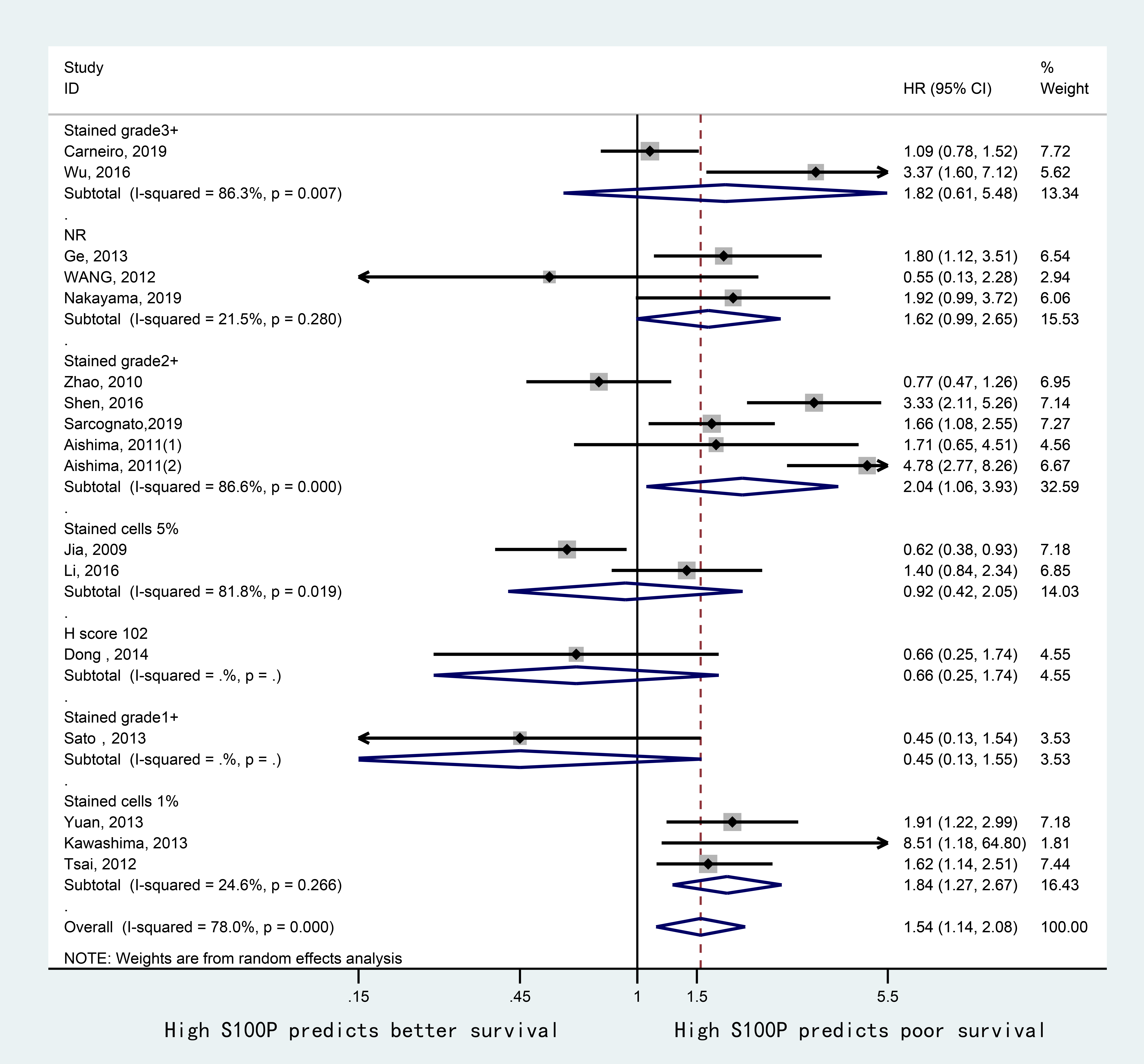

Supplement: Supplementary Figure 2 — Forest plots for the association between S100P expression and OS categorized by cut-off value. [file Image_2.tif]
