# Supplementary material for: Prognostic Value of S100P Expression in Patients With Digestive System Cancers: A Meta-Analysis
Source: Front Oncol. 2021 Mar 5;11:593728. doi: 10.3389/fonc.2021.593728 (PMC7973272; doi:10.3389/fonc.2021.593728)
Supplement: Supplementary file 3 [file Table_1.docx]

**Supplementary Materials: Table S1.** Pooled ORs for the relationship between high S100P expression level and clinicopathological parameters in gastric cancer, colorectal cancer, Cholangiocarcinoma (include Intrahepatic cholangiocarcinoma and extrahepatic bile duct carcinoma).

| **Tumor type** | **Clinicopathological feature** | **Number of studies** | **Number of patients** | **OR (95% CI)** | **P** | **Heterogeneity** | | |
| --- | --- | --- | --- | --- | --- | --- | --- | --- |
|  |  |  |  |  |  | **I^2^ (%)** | **P** | **Model** |
| Gastric cancer | Gender (male vs female) | 2 | 439（267/172） | 0.60 (0.39-0.92) | 0.019 | 40.3 | 0.196 | Fixed |
|  | Lymph node metastasis  (yes vs no) | 2 | 436(293/143) | 0.87 (0.56-1.37) | 0.556 | 0.0 | 0.473 | Fixed |
| Colorectal cancer | Gender (male vs female) | 3 | 312(190/122) | 1.26 (0.78-2.04) | 0.349 | 0.0 | 0.991 | Fixed |
|  | Differentiation (poor  vs moderate/well) | 3 | 312(50/262) | 0.97 (0.52-1.80) | 0.926 | 0.0 | 0.407 | Fixed |
|  | Lymph node metastasis  (yes vs no) | 2 | 216(96/120) | 2.47 (0.81-7.54) | 0.113 | 73.3 | 0.053 | Random |
|  | Tumor stage  (III/IV vs I/II) | 3 | 312(161/151) | 1.94 (0.59-6.37) | 0.274 | 83.4 | 0.002 | Random |
| Cholangiocarcinoma | Gender (male vs female) | 3 | 245(152/93) | 1.21 (0.50-2.93) | 0.671 | 57.5 | 0.095 | Random |
|  | Differentiation (poor  vs moderate/well) | 4 | 216(86/130) | 1.78 (0.89-3.56) | 0.105 | 0.0 | 0.809 | Fixed |
|  | Lymph node metastasis  (yes vs no) | 5 | 310(87/223) | 3.01 (1.63-5.56) | ＜0.001 | 29.9 | 0.222 | Fixed |
|  | Vascular invasion  (Present vs Absent) | 2 | 110(75/35) | 2.83 (0.24-33.91) | 0.412 | 58.2 | 0.122 | Random |
